# Doctor's Will

**Published:** 1913-12-13

**Authors:** 


### Doctor's Will.

Surgeon-Lieutenant-Colonel Henry Walter Kiallmark, M.R.C.S., of Ditchling, Sussex, late of the Royal Bucks Hussars and the Army Medical Reserve, and staff surgeon to the Ottoman Army, who left estate of the gross value of £17,156, left £1,350 to charitable institutions.

